# The Expression of the *fim* Operon Is Crucial for the Survival of *Streptococcus parasanguinis* FW213 within Macrophages but Not Acid Tolerance

**DOI:** 10.1371/journal.pone.0066163

**Published:** 2013-06-18

**Authors:** Yi-Ywan M. Chen, Hui-Ru Shieh, Ya-Ching Chang

**Affiliations:** 1 Department of Microbiology and Immunology, College of Medicine, Chang Gung University, Tao-Yuan, Taiwan; 2 Graduate Institute of Biomedical Sciences, College of Medicine, Chang Gung University, Tao-Yuan, Taiwan; University of Cambridge, United Kingdom

## Abstract

The acquisition of transition metal ions is essential for the viability and in some cases the expression of virulence genes in bacteria. The *fimCBA* operon of *Streptococcus parasanguinis* FW213 encodes a Mn^2+^/Fe^2+^-specific ATP-binding cassette transporter. FimA, a lipoprotein in the system, is essential for the development of endocarditis, presumably by binding to fibrin monolayers on the damaged heart tissue. Recent sequence analysis revealed that Spaf_0344 was homologous to *Streptococcus gordonii scaR*, encoding a metalloregulatory protein for the Sca Mn^2+^-specific transporter. Based on the homology, Spaf_0344 was designated *fimR*. By using various *fim* promoter (p*_fim_*) derivatives fused with a promoterless chloramphenicol acetyltransferase gene, the functions of the *cis*-elements of p*_fim_* were analyzed in the wild-type and *fimR*-deficient hosts. The result indicated that FimR represses the expression of p*_fim_* and the palindromic sequences 5′ to *fimC* are involved in repression of p*_fim_*. A direct interaction between FimR and the palindromic sequences was further confirmed by *in vitro* electrophoresis gel mobility shift assay and *in vivo* chromatin immunoprecipitation assay (ChIP)-quantitative real-time PCR (qPCR). The result of the ChIP-qPCR analysis also indicated that FimR is activated by Mn^2+^ and, to a lesser degree, Fe^2+^. Functional analysis indicated that the expression of FimA in *S. parasanguinis* was critical for wild-type levels of survival against oxidative stress and within phagocytes, but not for acid tolerance. Taken together, in addition to acting as an adhesin (FimA), the expression of the *fim* operon is critical for the pathogenic capacity of *S. parasanguinis*.

## Introduction

*Streptococcus parasanguinis* is a primary colonizer of the tooth surface and an important member of the dental plaque [1], [2]. Occasionally, *S. parasanguinis* and other viridians streptococci can enter the bloodstream, causing a transient bacteremia and infective endocarditis on native and prosthetic heart valves [3], [4]. Although the significance of *S. parasanguinis* in the oral ecosystem and systemic infection is well established, thus far the only known virulence factor associated with endocarditis is FimA of the FimCBA Mn^2+^/Fe^3+^ ATP-binding cassette (ABC) transporter [5]. FimA, a member of the lipoprotein receptor antigen I (LraI) family, participates in both metal transportation [5] and adherence to fibrin [6]. Binding to the fibrin and platelets deposited on the damaged heart tissues is critical for vegetation formation; therefore, it is proposed that FimA mediates the development of endocarditis by binding to the fibrin monolayer [6]. A FimA-deficient *S. parasanguinis* is avirulent in an animal model [6]. Immunization with the purified FimA protein prior to infection with *S. parasanguinis* FW213 also reduces the frequency and severity of infection in the rat model [7], further confirming the impact of FimA in disease development.

Genes encoding FimCBA transporter along with *tpx*, encoding a thiol peroxidase, are arranged as an operon in *S. parasanguinis* FW213 (*fimCBA-tpx*) [8]. The expression of the promoter located 5′ to *fimC* (p*_fim_*), which transcribes *fimCBA* and *tpx* [5], [8], is inhibited by 10 µM Mn^2+^, but neither Fe^3+^ nor Mg^2+^ influences the expression [5]. An additional constitutive promoter, p*_tpx_*, is located in the intergenic region between *fimA* and *tpx* [8], thus the expression of *tpx* is initiated from both p*_fim_* and p*_tpx_* [8], [9].

The *fimCBA-tpx* operon arrangement of *S. parasanguinis* is similar to the *psa* operon of *Streptococcus pneumoniae*, the *ssa* operon of *Streptococcus sanguinis*, and the *sca* operon of *Streptococcus gordonii* [10]–[12]. A homologous operon, *sloABCR*, is present in *Streptococcus mutans* [13]. However, instead of a *tpx*, the last gene of the *slo* operon encodes a metalloregulatory protein for the Slo system, whereas the loci encoding the specific regulators of the Psa, Ssa and Sca systems are not located in the flanking region of the structural genes [14], [15]. In addition, FimA, along with PsaA of the Psa system, SsaB of the Ssa system, ScaA of the Sca system, and SloC of the Slo system all play a major role in the virulence capacity of the microbes [10], [12], [15]–[21].

The expression of *psa*, *sca* and *slo* operons is subject to the regulation of PsaR, ScaR and SloR of the Diphtheria toxin repressor (DtxR) family, respectively, in the presence of excess amounts of cognate metal ions [14], [22], [23]. The consensus binding sequence of DtxR and its homologues has been determined as the key *cis*-regulatory element in several systems [15], [24]. The binding sequences of PsaR, ScaR and SloR all contain a palindromic sequence rich in A/T, albeit the overall lengths of the proposed operators vary among the three. Specifically, the predicted operators for PsaR [22] and SloR [25] contain one palindrome of an 8-nucleotide (nt) inverted repeat spaced by 6 nt (AAAATTAACTTGACTTAATTTT), whereas the proposed operator of ScaR contains an additional imperfect inverted repeat of 9 nt (TGTTAAGGTATATTAATA), with a total length of 46 nt [14]. Although the second inverted repeat was also observed in *psa* and *slo* promoters with a distance to the first palindrome similar to that in *sca* operon, the function of the second palindrome in the binding of PsaR and SloR is unknown. Both PsaR and ScaR are activated by Mn^2+^ and additional metal ions, such as Cd^2+^, but not Zn^2+^, and it is suggested that an excess amount of Zn^2+^ could ensure an optimal uptake of Mn^2+^ by inactivation of PsaR and ScaR [26], [27]. On the other hand, SloR is a bifunctional regulator that exerts both positive and negative regulation when Mn^2+^ is available. SloR is a repressor if the SloR recognition element (SRE) is located within 50 bp of the transcription initiation site of the target gene. When the SREs are located further upstream, SloR acts as an activator [25]. Moreover, like many other metalloregulatory proteins, both PsaR and SloR regulate other genes in addition to the cognate metal uptake system [22], [25], confirming the critical role of the intracellular metal homeostasis in the physiology and pathogenesis.

A *scaR* homologue (Spaf_0344), approximately 2 kbp 3′ to the *fim* operon, was identified previously by chromosomal walking (ACR24649). The recent transcriptomic analysis of *S. parasanguinis* FW213 further confirmed the expression of Spaf_0344 [28]. In this study, we investigated the regulatory function of Spaf_0344 on *fim* operon expression, and the impact of the regulation on the pathogenic capacity of *S. parasanguinis* FW213. Our results indicated that in addition to acting as an adhesin (FimA), the expression of the *fim* operon in *S. parasanguinis* is critical for the optimal capacity against oxidative stress and wild-type levels of survival within phagocytes.

## Results

### Identification of *fimR*

**Figure 1:**
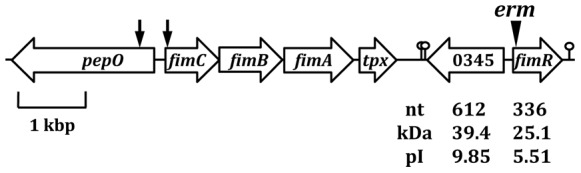
Schematic diagram of the *fim* operon and its flanking regions of *S.* *parasanguinis* FW213. The relative location and transcription direction of each ORF are shown. Spaf_0345 and Spaf_0344 are indicated as 0345 and *fimR*, respectively. The limits of the sequence present in Figure 2A are indicated by two vertical arrows. The position of the *erm* in strain Δ*fimR* is indicated by an inverted triangle above the gene. The putative terminators for Spaf_0345 and *fimR* are indicated. The sizes of Spaf_0345 and *fimR* in nt, predicted molecular weight in kDa and pI of the gene products are shown.

Sequence analysis of the 3′ flanking region of *fim* operon revealed two open reading frames (ORFs), Spaf_0345 and Spaf_0344, in opposite orientations (Figure 1). Both ORFs began with an ATG translation start codon and were preceded by a putative ribosomal binding site (RBS). Two putative rho-independent terminators were located at 19 bp (ΔG° = −4.7 kcal mol^−1^) and 52 bp (ΔG° = −4.2 kcal mol^−1^) 3′ to the stop codon of Spaf_0345, respectively. An inverted repeat (ΔG° = −1.9 kcal mol^−1^) was found 33 bp 3′ to the stop codon of Spaf_0344. The deduced amino acid (aa) sequence of Spaf_0345 shares significant homology with a hypothetical protein of *Streptococcus australis* ATCC 700641 (HMPREF9961_1041, 74% identity) and *S. sanguinis* SK36 (SSA_0258, 41% identity). Additional homologues were found in *S. mutans* UA159 (SMU.741, 38% identity) and *Streptococcus agalactiae* 2603V/R (SAG0713, 35% identity). Thus far no functional analysis of Spaf_0345 is available. Of note, the expression of Spaf_0345 was evident by reverse transcription (RT)-PCR analysis, albeit the expression level of Spaf_0345 is only approximately 15% of that of *dnaA* in cells at mid-exponential growth phase (data not shown). Spaf_0344 shares significant homology at the deduced aa level with ScaR (65% identity) of *S. gordonii* CH1 (AF182402_1) and with SloR (55% identity) of *S. mutans* UA159 (NP_720655.1). Both ScaR and SloR belong to the DtxR family proteins, which are composed of an N-terminal helix-turn-helix (HTH) motif, followed by a metal binding and dimerization domain. Both regions are present in Spaf_0344, and the conserved His-79, Glu-83 and His-98 in the metal binding region I of DtxR [29] all were found in the corresponding locations in Spaf_0344. On the other hand, among the conserved residues in the metal binding region II of DtxR, only His-106 was found in Spaf_0344. The expression level of Spaf_0344 is comparable with that of *dnaA* in the exponential growth phase (data not shown). As ScaR participates in the expression regulation of *S. gordonii sca* operon [14], an ortholog of the *S. parasanguinis* FW213 *fim* operon, Spaf_0344 was designated *fimR*.

### FimR Negatively Regulated the Expression of p*_fim_*

**Figure 2:**
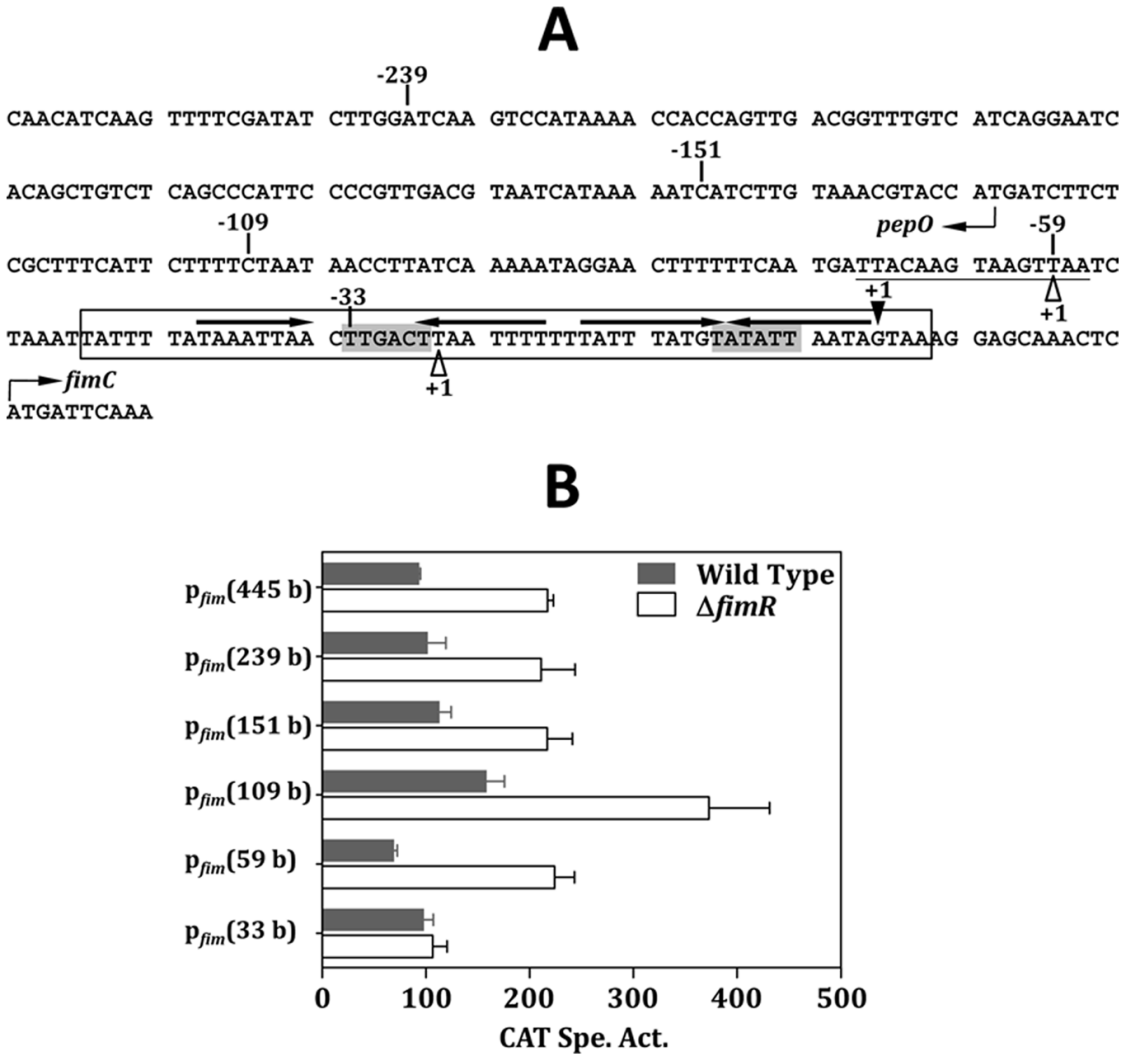
The regulation of FimR on p*_fim_* expression. (A) The nt sequence of the 5′ flanking region of *fimC*. The *pepO* and *fimC* are transcribed from the opposite DNA strands, thus the sequence of *pepO* presented here is the noncoding strand, and the sequence of *fimC* is the coding strand. The transcription initiation sites (+1) of *fimC* and *pepO* are shown by a solid triangle above the sequence, and two open triangles below the sequence, respectively. The putative −10 and −35 sequences of p*_fim_* are shaded. The potential Per box is underlined. The inverted repeat sequences are shown by horizontal arrows above the sequence. The sequence of the probe used in EMSA is boxed. The limits of the deletion derivatives are indicated by the numbers. (B) The CAT activities in wild-type FW213 and Δ*fimR* harboring various p*_fim_*-*cat* fusions. All strains were grown in TH. Values shown are means and standard deviations of three independent experiments. All experiments were done in triplicate reactions and negative controls were reactions carried out in the absence of Cm.

Sequence analysis reveals two inverted repeats in the intergenic region of *pepO* and *fimC* (Figure 2A), the potential targets of the DtxR-family proteins [14]. To analyze the impact of FimR on *fim* operon expression and its possible binding region, a series of p*_fim_-*chloramphenicol (Cm) acetyltransferase gene (*cat*) fusion derivatives with different lengths of the 5′ flanking region were established in the wild-type and *fimR*-deficient (Δ*fimR*) *S. parasanguinis* as detailed in the materials and methods. Of note, all fusions were tagged with a spectinomycin (Sp) resistance gene (*spe*) [30] at the 5′ end of the fusion. The *spe* cassette contains a strong terminator and is in the same transcription direction as the fusion, thus preventing any possible read through effect from the 5′ flanking region. Since regulatory proteins of DtxR family are generally activated by multiple metal ions, the promoter activity in all strains was determined in cells grown in the complex medium, Todd-Hewitt (TH) broth. A basal and unregulated Cm acetyltransferase (CAT) expression was observed in strains with the p*_fim_*(33 b)-*cat* fusion (Figure 2B). With all other fusion constructs, a lower level of CAT activity was detected in the wild-type background than that in Δ*fimR* (*P*<0.01, Student’s *t* test), indicating that FimR represses the expression of p*_fim_*. A comparable expression level was detected in strains harboring p*_fim_*(445 b)-*cat*, p*_fim_*(239 b)-*cat* and p*_fim_*(151 b)-*cat* fusions, indicating that all *cis*-elements are located within the 151-base region. As p*_fim_*(445 b)*-cat* fusion contains the longest 5′ flanking region of p*_fim_*, this fusion was used as the full-length p*_fim_* control in the following analysis. Elevated CAT activities were detected in both the wild-type FW213 and Δ*fimR* harboring the p*_fim_*(109 b)-*cat* fusion, suggesting that the sequence between −151 and −109 contains a negative regulatory element. Further shortening the length of p*_fim_* by 50 bases (p*_fim_*[59 b]-*cat*) reduced the CAT expression, suggesting that the sequence from −109 to −59 is essential for optimal expression. *Trans*-complementation of *fimR* on pDL276 (pHR6) in strain Δ*fimR* harboring p*_fim_*(445 b)-*cat* restored wild-type p*_fim_* expression (Figure S1). Taken together, p*_fim_* is negatively regulated by FimR. The different expression levels in Δ*fimR* harboring various fusions also suggest the presence of additional regulators.

### The Expression of p*_fim_* was Modulated by both Mn^2+^ and Fe^2+^

**Figure 3:**
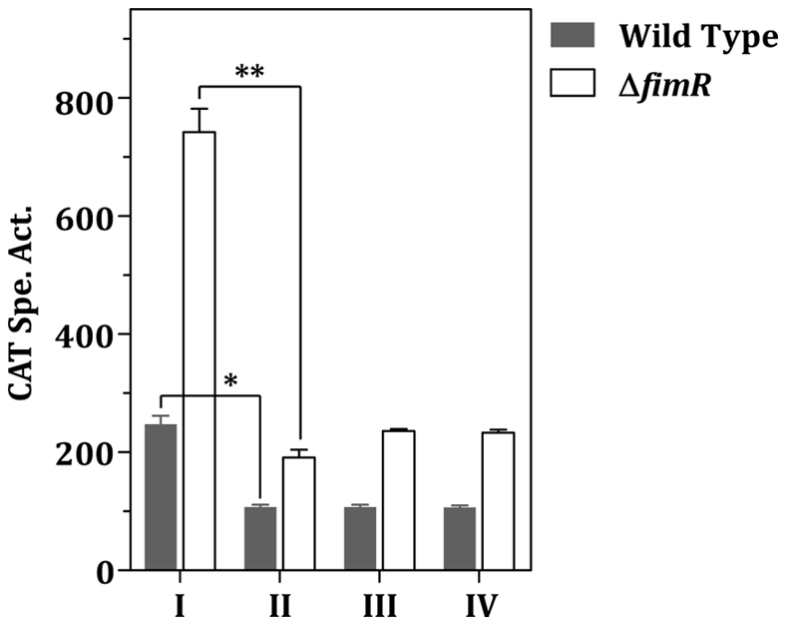
Effect of Mn^2+^ and Fe^2+^ on p*_fim_* expression. Wild-type FW213 and Δ*fimR* harboring p*_fim_*(445 b)-*cat* were grown in FMC containing 0.01 µM MnCl_2_ and 0.1 µM FeSO_4_ (I), 0.01 µM MnCl_2_ and 50 µM FeSO_4_ (II), 50 µM MnCl_2_ and 0.1 µM FeSO_4_ (III), 50 µM MnCl_2_ and 50 µM FeSO_4_ (IV). All cultures were supplemented with 1 mM MgSO_4_ and 1 mM CaCl_2_. Values are means and standard deviations of three independent experiments. Significant differences between samples were determined by two-way ANOVA using SPSS Statistic 17.0. The *P* values between the wild-type strain and Δ*fimR* under all four conditions are less than 0.01. *P* values between condition I and II are indicated in the figure. *, *P*<0.05; **, *P*<0.01.

Previous studies by Oetjen et al. demonstrated that *fimCBA* encodes an uptake system for manganese and iron [5]. However, the expression of the *fim* operon is repressed only by Mn^2+^ but not Fe^3+^. To investigate whether Fe^2+^ is involved in the FimR-mediated regulation, the CAT activity in the wild-type FW213 and Δ*fimR* in the presence of various amounts of Mn^2+^ and Fe^2+^ was determined (Figure 3). To precisely control the content of the metal ions, cells were cultivated in the chemically-defined medium FMC supplemented with various amounts of metal ions as detailed in the materials and methods. As expected, an up regulation of p*_fim_* expression was consistently observed in Δ*fimR* under all conditions used, confirming the negative effect of FimR on p*_fim_* expression. With 50 µM of Mn^2+^ and/or Fe^2+^, the p*_fim_* activity in Δ*fimR* was approximately twofold higher than that in the wild-type strain, and comparable expression levels were detected among the three conditions (Figure 3, lanes II to IV), indicating that FimR is active in the presence of Mn^2+^ or Fe^2+^. However, when cells were grown under limited Mn^2+^ (0.01 µM) and Fe^2+^ (0.1 µM), a further up regulation was observed in both the wild-type and Δ*fimR* hosts (Figure 3, lane I), suggesting that additional regulation modulated by the amounts of Mn^2+^ and Fe^2+^ also participates in the regulation. To confirm that the observed differential expression in response to Mn^2+^ and Fe^2+^ was driven by the promoter but not the nature of CAT, we also monitored the activity of p*_fap1_*, whose expression is insensitive to Mn^2+^ and/or Fe^2+^ contents, under various metal conditions by using a *S. parasanguinis* p*_fap1_*-*cat* fusion stain. A comparable CAT activity was observed in this strain under all four metal conditions (Figure S2), confirming the regulation of p*_fim_* in response to metal conditions.

### FimR Binds to p*_fim_* Directly

**Figure 4:**
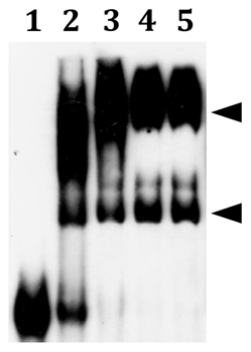
EMSA demonstrating the interaction between FimR and p***_fim_*** **.** Lanes 1 to 4 are reactions containing 0, 20, 40, and 80 µM His-FimR, respectively; lane 5 is reaction containing 80 µM His-FimR and unlabeled *tcrB*. The positions of the FimR-probe complexes are indicated by triangles.

As the predicted FimR binding site overlaps with −35 and −10 elements of p*_fim_*, the reporter assay described above does not allow us to analyze directly the impact of this region in p*_fim_* expression. Thus, electrophoretic mobility shift assay (EMSA) was used to determine if FimR directly interacts with p*_fim_*. A biotin-labeled DNA fragment containing both inverted repeats 5′ to the +1 of p*_fim_* (Figure 2A) was incubated with increasing amounts of purified histidine-tagged FimR (His-FimR) in the presence Mn^2+^. Two probe-FimR complexes were evident with 40 µM His-FimR, and the complexes with the slower mobility become clear in the reaction with 80 µM His-FimR (Figure 4). The shift pattern remained the same in the presence of additional unlabelled *tcrB* fragment, indicating that FimR binds specifically to the p*_fim_* probe. This result also suggests the presence of two FimR binding sites on the probe.

**Figure 5:**
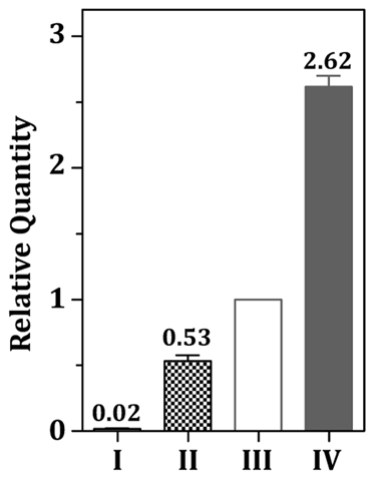
ChIP-qPCR demonstrating the relative quantity of p*_fim_* bound by FimR. Cells were grown under 0.01 µM MnCl_2_ and 0.1 µM FeSO_4_ (I), 0.01 µM MnCl_2_ and 50 µM FeSO_4_ (II), 50 µM MnCl_2_ and 0.1 µM FeSO_4_ (III), and 50 µM MnCl_2_ and 50 µM FeSO_4_ (IV). The ΔCq of the sample from III was used as the reference. Significant differences between samples were determined using one-way ANOVA. A significant difference (*P*<0.05) was detected between all pairs of comparison.

To confirm the *in vivo* binding of FimR to p*_fim_*, and to determine the impact of Mn^2+^ and Fe^2+^ on the binding activity of FimR, chromatin immunoprecipitation (ChIP) assay-quantitative real time PCR (qPCR) with anti-FimR antibody was employed as detailed in the materials and methods. The strongest binding of FimR to p*_fim_* was detected in cells grown in the presence of 50 µM MnCl_2_ and 50 µM FeSO_4_ (Figure 5, lane IV), whereas minimal amounts of MnCl_2_ (0.01 µM) and FeSO_4_ (0.1 µM) led to the weakest binding (Figure 5, lane I). Although both Fe^2+^ and Mn^2+^ at 50 µM can activate FimR, an 1.8-fold increase in the relative quantity was observed when 50 µM Mn^2+^ was provided in the culture medium compared to medium containing 50 µM Fe^2+^ (Figure 5, lanes II and III), indicating that Mn^2+^ is more effective than Fe^2+^ for FimR activation. Taken together, in the presence of excess amounts of Mn^2+^ or Fe^2+^, FimR repressed the expression of p*_fim_* by directly binding to the target sequence.

### FimA is Required for *S. parasanguinis* Defense against Oxidative Stress

**Figure 6:**
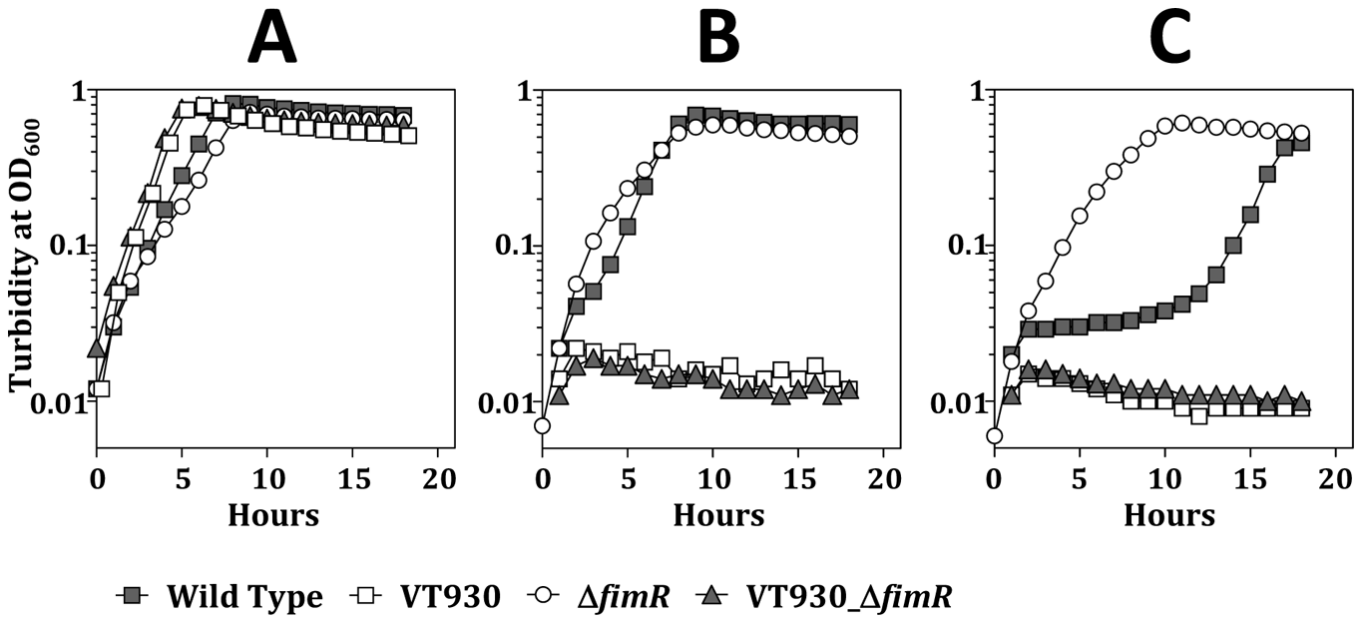
Growth kinetics of the wild-type *S.* *parasanguinis*, VT930, Δ*fimR*, and VT930_Δ*fimR* grown in TH (A), TH containing 2 mM (B) and 4 mM (C) paraquat. A representative figure of at least three experiments under each condition is shown.

As intracellular metal homeostasis is linked closely to the oxidative stress response, the possible role of FimA and FimR regulation in avoiding oxidative challenge was examined. Generally, regulatory proteins of DtxR family modulate not only metal homeostasis but also the expression of other genes, thus a *fimA* and *fimR* double mutant strain (VT930_Δ*fimR*) was also included in the following studies to differentiate the impact of *fimA* and other genes regulated by FimR. The growth of all strains in the presence of paraquat, a redox-cycling compound that can cause oxidative stress by generating superoxide radical in the cytoplasm, was monitored. It was noticed that inactivation of *fimA* (VT930) [31] or both *fimA* and *fimR* (VT930_Δ*fimR*) enhanced the growth in TH broth, whereas *fimR*-deficiency alone (Δ*fimR*) led to a longer doubling time than the wild-type strain (Figure 6A). The estimated doubling time for the wild-type FW213, VT930, Δ*fimR* and VT930_Δ*fimR* in TH is 80, 50, 105 and 50 min, respectively. In the presence of 2 mM paraquat, a reduced growth rate was detected in both the wild-type FW213 and Δ*fimR*. The lag phase in Δ*fimR* was slightly shorter than that in the wild-type strain in the presence of 2 mM paraquat (Figure 6B), and the difference between these two strains was more pronounced under 4 mM paraquat (Figure 6C). On the other hand, the growth of VT930 and VT930_Δ*fimR* was severely hampered in the presence of paraquat. Thus, a functional FimCBA transport system is essential for optimal oxidative stress responses in *S. parasanguinis*. As VT930 and VT930_Δ*fimR* bear a similar capacity against paraquat challenge, it is concluded that the expression of *fimA* plays a key role in this process.

### FimA Enhances the Intracellular Survival of *S. parasanguinis* within Macrophages

**Figure 7:**
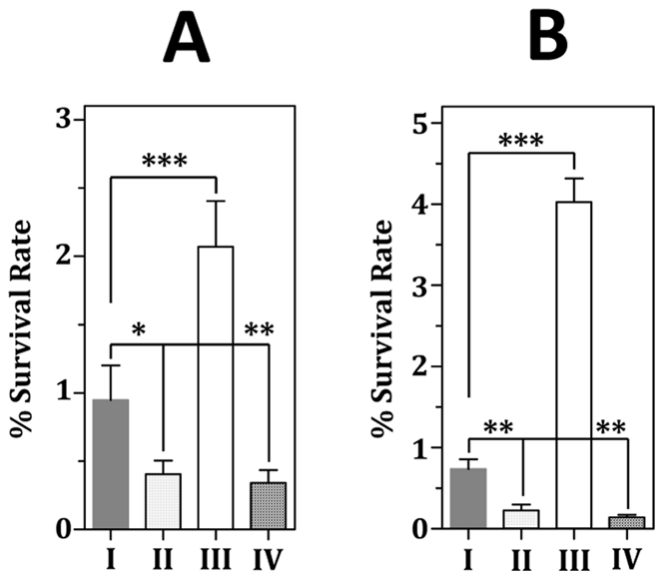
The survival rate of the wild-type *S.* *parasanguinis* (I), VT930 (II), Δ*fimR* (III), and VT930_Δ*fimR* (IV) in THP1 (A) and RAW264.7 (B). The numbers are means and standard deviations of three independent experiments. All experiments were done with triplicate samples. Significant differences between wild-type and recombinant strains were analyzed by one-way ANOVA. ***, *P*<0.01; **, *P*<0.05; *, *P*<0. 1.

Macrophages are critical for defending microbial infection, thus the impact of FimA and FimR regulation in the survival of *S. parasanguinis* within macrophages was analyzed. Of note, inactivation of *fimA* does not inhibit the uptake of the bacteria by granulocytes [6]. The intracellular survival rate of VT930 and VT930_Δ*fimR* within THP1 was less than 50% of that of wild-type FW213, whereas the survival rate of Δ*fimR* was twofold greater than that of wild-type FW213 (Figure 7A). A similar survival pattern between wild-type FW213, VT930, Δ*fimR* and VT930_Δ*fimR* was detected in RAW264.7 macrophages (Figure 7B). As the expression of *fim* operon was negatively regulated by FimR, and VT930 and VT930_Δ*fimR* exhibited a comparable survival rate in both macrophages used, these results indicated that the expression of FimA is critical for wild-type levels of survival within macrophages.

### The Expression of the *fim* Operon is not Required for the Acid Tolerance of *S. parasanguinis*

**Figure 8:**
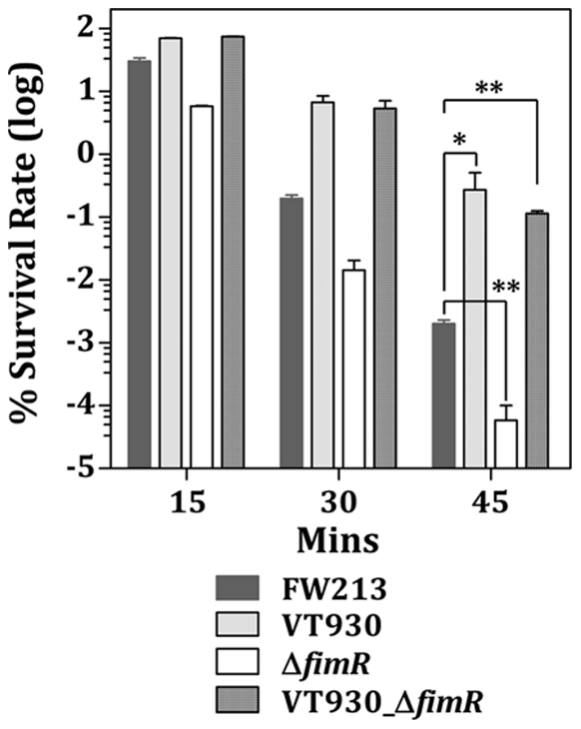
Acid killing assay. The means and standard deviations for three independent samples are shown. Significant differences between the wild-type and recombinant strains at 45 min were analyzed by one-way ANOVA. **, *P*<0.05; *, *P*<0. 1.

As oxidative stress responses are known to overlap with acid tolerance [32]–[35], the possible function of FimA and FimR in acid tolerance was determined by an acid killing assay. When the survival rates at pH 3 was examined, a time-dependent decline in survival rate was observed with all strains tested. Interestingly, the viability of Δ*fimR* was lower than that of the wild-type FW213, whereas inactivation of *fimA* or both *fimA* and *fimR* enhanced the survival at pH 3 (Figure 8). These results indicated that, opposite to the oxidative stress responses, *S. parasanguinis* was more sensitive to acidic challenges when the *fim* operon was highly expressed.

## Discussion

This study set to investigate the regulation and expression of the FimCBA transport system on the pathogenic capacity of *S. parasanguinis* FW213. We found that the expression of the *fim* operon is regulated by FimR and additional *trans*-acting element(s), and the expression of the *fim* operon is critical for the oxidative stress responses and survival of *S. parasanguinis* against phagocytic killing. Our results also indicated that the expression of p*_fim_* is sensitive to both Mn^2+^ and Fe^2+^. Such regulation will ensure an adequate uptake of Mn^2+^ and Fe^2+^ for growth and avoid potential toxicity caused by excess amounts of intracellular Fe^2+^. As homologues of FimR are known global regulators, it is likely that *S. parasanguinis* possesses a FimR regulon. However, all phenotypes of Δ*fimR* observed in this study result from up regulation of the *fim* operon, indicating that the intracellular homeostasis of Mn^2+^ and Fe^2+^ is critical for the described phenotypes.

That metal uptake systems are regulated by multiple regulators is not unique to the *S. parasanguinis* FimCBA system. For instance, the expression of the *mtsABC* of *Streptococcus pyogenes*, encoding an ABC transporter for Mn^2+^ (mainly) and Fe^3+^, is regulated by both MtsR and PerR [36]. Both MtsA and FimA belong to the LraI family [37], and MtsR, a member of the DtxR family proteins, represses the expression of *mtsABC* in response to Mn^2+^. PerR, a paralogue of Fur, generally acts as a metal-dependent and oxidative-responsive repressor. However, PerR positively regulates the expression of *mtsABC* unresponsive to Mn^2+^, Fe^3+^, and Zn^2+^ in *S. pyogenes* [36], [38]. Sequencing analysis of the 5′ flanking region of *S. parasanguinis fimC* revealed a putative Per box located at −71 to −57 of p*_fim_* (Figure 2A). This motif differs from the consensus sequence (TTANAATNATTNTAA) derived from *Bacillus subtilis* and *S. pyogenes* [39] by 3 bases (Figure 2A). Furthermore, the BlastX search result found that Spaf_616 encodes a Fur family transcriptional regulator that shares 83% identity with the PerR of *S. pyogenes* MGAS6180 (AAX71273). As a positive effect on expression was detected between −109 to −59 of p*_fim_*, it is possible that the expression of the *fim* operon in *S. parasanguinis* is positively regulated by Spaf_616. Unfortunately, multiple tries failed to generate a Spaf_616 mutant, thus the possible involvement of Spaf_616 in *fim* operon expression remains unknown.

It is peculiar that the highest p*_fim_* activity was detected in strain p*_fim_*(109 b)-*cat*, whereas both extending and reducing the promoter by 42 b (p*_fim_*[151 b]-*cat*) and 50 b (p*_fim_*[59 b]-*cat*), respectively, reduced the promoter activity (Figure 2C). Further sequence analysis revealed a putative catabolite responsive element (*cre*) (TGTAAACGTACCAT), the binding sequence of the catabolic control protein A (CcpA), located at −146 to −133 of p*_fim_*. This motif is only 2 bases different from the proposed *cre* of *S. pyogenes* (TGWAANSBHTWHHW) [40]. Interestingly, inactivation of *ccpA* led to a higher CAT activity in FW213 harboring p*_fim_*(151 b)-*cat*. However, the increase in CAT activity was also detected in strains p*_fim_*(109 b)-*cat* and p*_fim_*(59 b)-*cat* (data not shown), indicating that the predicted *cre* is not involved in the regulation and CcpA modulates p*_fim_* expression indirectly. As the FimCBA transport system also transports iron, presumably the CcpA-mediated repression of p*_fim_* could provide an additional control of the intracellular iron and subsequently reduce the oxidative damage resulting from the Fenton reaction. The link between metabolism and oxidative stress response via the regulation of CcpA has been reported in *Lactococcus lactis* [41]. CcpA activates the expression of FhuR, the repressor for the haem uptake system FhuBGD, and thus prevents oxidative damage caused by excess amounts of intracellular iron at the onset of exponential growth in *L. lactis* [41]. Of note, no potential *cre* was detected in the 5′ flanking region of *fimR*, thus, the function of CcpA on p*_fim_* remains unclear.

The intracellular manganese and zinc homeostasis are co-regulated by PsaR and AdcR in *S. pneumoniae* [26]. AdcR is the repressor of the AdcCBA Zn^2+^ ABC transporter that represses the expression of *adcCBA* in the presence of excess amounts of Zn^2+^ [42]. Excess amounts of intracellular Zn^2+^ resulted from *adcR*-deficiency can compete with Mn^2+^ in binding to PsaR, albeit at a lower efficiency, and subsequently derepress *psa* operon [26]. An *adcRCBA* homologue is present in the genome of *S. parasanguinis* FW213. However, in contrast to the regulation in *S. pneumoniae*, inactivation of *adcR* with a non-polar *erm* lowered p*_fim_* expression in *S. parasanguinis*, regardless the amount of Zn^2+^ in the growth medium (data now shown), indicating that AdcR positively regulates p*_fim_* expression. As we did not observe any potential AdcR binding sequence in the 5′ region of *fimC*, nor did we detect any interaction between AdcR and p*_fim_* DNA fragment in EMSA (data now shown), it is more likely that AdcR binds to a yet-to-be-identified protein and regulates p*_fim_* indirectly.

The generation of reactive oxygen species and reactive nitrogen species by activated immune cells is essential for animal and plant innate immune defenses against invading pathogens. It has also been suggested that phagocytes control the replication of invading bacteria within phagosomes partially via the activity of *n*atural *r*esistance-*a*ssociated *m*acrophage *p*rotein (Nramp1), which catalyzes the efflux of divalent cations in a H^+^-dependent manner [43]. Mn^2+^ is an important cofactor for several bacterial enzymes, including the Mn^+^-dependent superoxide dismutase (MnSOD), and enzymes participating in carbon metabolism and stringent response [44], therefore an elevated Mn^2+^ uptake capacity, as seen in Δ*fimR*, will enhance the survival of *S. parasanguinis* within phagocytes. Although we could not rule out the possibility that additional genes/operons regulated by FimR may also contribute to the survival of *S. parasanguinis* within phagocytes, the impact of FimA in this process is very clear.

Studies by Bruno-Bárcena revealed that activation of MnSOD can enhance the resistance of *Streptococcus thermophilus* against acid stress by reducing the frequency of the intracellular iron-mediated oxidative stress [35]. Such regulation also suggests that a low intracellular iron concentration coincides with optimal acid tolerance. As the content of iron in brain-heart-infusion-based medium is approximately 100-fold higher than that of manganese [26], it is possible that inactivation of *fimR* could lead to an increased intracellular concentration of iron over manganese via the transportation of the FimCBA system, and subsequently enhanced Fenton reaction and reduced survival at pH 3. On the other hand, inactivation of *fimA* would result in a minimal amount of intracellular Mn^2+^/Fe^2+^ and enhanced acid tolerance.

### Conclusions

In conclusion, this study demonstrated that the expression of the *fim* operon in *S. parasanguinis* provides protection against phagocytic killing. Over expression of this system disrupts the acid survival in *S. parasanguinis*, presumably via an enhanced intracellular Fenton reaction. The complexity of the p*_fim_* regulation suggests that an optimal expression of the *fim* operon is critical for the survival of *S. parasanguinis*.

## Materials and Methods

### Bacteria Strains, Plasmids, Culture Media and Growth Conditions

*S. parasanguinis* FW213 [45] and its derivatives were cultivated routinely in TH broth at 37°C in a 10% CO_2_ atmosphere. Where indicated, spectinomycin (Sp) at 500 µg ml^−1^, erythromycin (Em) at 5 µg ml^−1^, or kanamycin (Km) at 200 µg ml^−1^ were included in the media for maintaining recombinant *S. parasanguinis* strains. To analyze the effects of metal ions on growth, the chemically defined medium FMC [46] was used with modifications. Where indicated, the FMC was treated with Chelex-100 (Sigma, United States) at 55°C for 24 h to remove all divalent metal ions. The essential metal ions were then refurnished by the addition of 0.01 or 50 µM MnCl_2_, 0.1 or 50 µM FeSO_4_, 1 mM MgSO_4_, and 1 mM CaCl_2_. Recombinant *E. coli* strains were routinely cultured in LB broth containing ampicillin (Ap) at 100 µg ml^−1^, Km at 50 µg ml^−1^, Em at 200 µg ml^−1^, or Cm at 25 µg ml^−1^ as needed. The bacterial strains and plasmids used in this study are listed in Table 1.

**Table 1:** Bacterial strains and plasmids used in this study.

Strain or Plasmid	Relevant phenotypes^a^	Description	Source
**Strains**			
*S. parasanguinis*			
FW213		Wild-type strain	[45]
p*_fim_*(33 b)-*cat*	Sp^r^	FW213 harboring p*_fim_*(33 b)-*cat* at *tcrB*	This study
p*_fim_*(59 b)-*cat*	Sp^r^	FW213 harboring p*_fim_*(599 b)-*cat* at *tcrB*	This study
p*_fim_*(109 b)-*cat*	Sp^r^	FW213 harboring p*_fim_*(109 b)-*cat* at *tcrB*	This study
p*_fim_*(151 b)-*cat*	Sp^r^	FW213 harboring p*_fim_*(151 b)-*cat* at *tcrB*	This study
p*_fim_*(239 b)-*cat*	Sp^r^	FW213 harboring p*_fim_*(239 b)-*cat* at *tcrB*	This study
p*_fim_*(445 b)-*cat*	Sp^r^	FW213 harboring p*_fim_*(445 b)-*cat* at *tcrB*	This study
VT930	Km^r^, FimA^−^	FW213 *fimA*::*aphA3*	[31]
VT930_Δ*fimR*	Km^r^, Em^r^, FimA^−^, FimR^−^	VT930 containing a deletion in *fimR*	This study
ΔR_p*_fim_*(33 b)-*cat*	Sp^r^, Em^r^, FimR^−^	*fimR*-deletion mutant harboring p*_fim_*(33 b)-*cat* at *tcrB*	This study
ΔR_p*_fim_*(59 b)-*cat*	Sp^r^, Em^r^, FimR^−^	*fimR*-deletion mutant harboring p*_fim_*(59 b)-*cat* at *tcrB*	This study
ΔR_p*_fim_*(109 b)-*cat*	Sp^r^, Em^r^, FimR^−^	*fimR*-deletion mutant harboring p*_fim_*(109 b)-*cat* at *tcrB*	This study
ΔR_p*_fim_*(151 b)-*cat*	Sp^r^, Em^r^, FimR^−^	*fimR*-deletion mutant harboring p*_fim_*(151 b)-*cat* at *tcrB*	This study
ΔR_p*_fim_*(239 b)-*cat*	Sp^r^, Em^r^, FimR^−^	*fimR*-deletion mutant harboring p*_fim_*(239 b)-*cat* at *tcrB*	This study
ΔR_p*_fim_*(445 b)-*cat*	Sp^r^, Em^r^, FimR^−^	*fimR*-deletion mutant harboring p*_fim_*(445 b)-*cat* at *tcrB*	This study
Δ*fimR*/pHR6	Sp^r^, Em^r^, Km^r^	Strain ΔR_p*_fim_*(445 b)-*cat* harboring pHR6	This study
**Plasmids**			
pDL276	Km^r^	*Streptococcus-E. coli* shuttle vector	[52]
pGEM3Zf(+)	Ap^r^	General *E. coli* cloning vector	Promega
pHR3	Ap^r^, Sp^r^	pGEM3ZF(+)/*tcrB*::*spe-*p*_fim_*(445 b)-*cat*	This study
pHR6	Km^r^	pDL276/*fimR*	This study
pQE30	Ap^r^	Expression vector of His-tagged proteins	Qiagen
pQE30/fimR	Ap^r^	pQE30 harboring the coding sequence of *fimR*	This study
pSU21	Cm^r^	pACYC184-based *E. coli* cloning vector	[51]

a,r resistance; -, deficiency.

### General Genetic Techniques

Genomic DNA and total cellular RNA were isolated from *S. parasanguinis* as previously described [47], [48]. Plasmid DNA was isolated from recombinant streptococcal strains by the method of Anderson and McKay [49]. Plasmid DNA was introduced into *S. parasanguinis* and its derivatives via electroporation as previously described [48]. PCRs were carried out by using Vent® (NEB, United States) or Blend Taq*®* DNA polymerase (TOYOBO, Japan). All primers used in this study are listed in Table 2.

**Table 2:** Primers used in this study.

Primer	Sequence^a^
fimC/ASBamHI	GAATCATGGATCCGCTCCTTTACTATT
fimC/AS5025	GGATGGTTGGACCCTGGATGGTG
fimC_5′/S4731	GCTGTCTCAGCCCATTCCCCGTTGACG
fimR/AS9576SmaI	AAACCCGGGCTTCTTTGTTTGGTGTCATG
fimR/AS10331PstI	GAAGTCCCTGCAGAACGAGGGATCTTT
fimR/AS10915SacI	GCCGGAGCTCTACTCTGTTAAGCGTAC
fimR/S8023SpeI	AGGACTAGTACCTCTTTTCTATATCTAC
fimR/S9221BamHI	CATGAGGATCCTCAACAAGTCTTGGGC
fimR/S9761SmaI	AAACCCGGGTCTCTGATCTCTACCG
fimR/MSSacI	GAAGAAAAGAGGAGCTCATGACACC
fimR/stopASPstI	GAAAGCATGCACTGCAGTTAGACTGCA
pDS-1	ATCAAGTCCATAAAACCAC
pDS-2	CATCTTGTAAACGTACCATGATC
pDS-3	CTAATAACCTTATCAAAAATAGGAAC
pDS-4	TAATCTAAATTATTTTATAAATTAACTTG
pDS-5	TTGACTTAATTTTTTTATTTATGTATATTA
pepO/AS320SacI	GTACCGAGCTCGCTGGTATAGTCTT
pfimbox/AS	TTACTATTAATATACATAAATAAAAAAATTAAGTCAAGTTAATTTATAAAATA
pfimbox/S	TATTTTATAAATTAACTTGACTTAATTTTTTTATTTATGTATATTAATAGTAA
spe/AS	GCAACTGCAGATTGTTTTCTAAAATCTGATT
tcrB/AS	CTATTTCTAAGGCTTGGCGG
tcrB/S	TGGCGATGAAGTAATCGGGG

a inserted restriction recognition sites are underlined.

### Construction of the Recombination *S. parasanguinis* p*_fim_-cat* Fusion Strain and its Deletion Derivatives

The p*_fim_* fragment, containing the 445 bp 5′ to the transcription initiation site (+1) of p*_fim_* and the region from +1 to the translation start codon of *fimC* (p*_fim_*[445 b]), was amplified from *S. parasanguinis* by PCR using primer pair pepO/AS320SacI and fimC/ASBamHI. A *Sac*I and a *Bam*HI recognition site were incorporated in the two primers, respectively, to facilitate the cloning. The promoter region was subsequently ligated to the 5′ end of a promoterless *cat* from *Staphylococcus aureus* pC194 [50]. The p*_fim_*(445 b)*-cat* fusion was confirmed by sequencing analysis, and a *spe* cassette was subsequently cloned into the 5′ end of the correct fusion. To facilitate the integration into FW213 chromosome, an internal fragment of *tcrB*, encoding a copper-(or silver)-translocating P-type ATPase, was amplified by PCR with primers tcrB/S and tcrB/AS, and subsequently cloned into pGEM3Zf(+). The *spe*-p*_fim_-cat* fusion was then cloned into the *Eco*RV site within *tcrB* to generate pHR3. To generate p*_fim_* deletion derivatives, an inverse PCR approach was employed by using pHR3 as the template. Briefly, five sense primers, pDS-1, 2, 3, 4, and 5, starting at 239, 151, 109, 59 and 33 bases 5′ to the +1 of p*_fim_*, were paired with an antisense primer, spe/AS, and used in inverse PCRs. The PCR products were subsequently self-ligated and established in *E. coli*. The identity of each clone was confirmed by sequencing analysis. Plasmid pHR3 and the 5 derivatives were introduced into *S. parasanguinis*, and the correct double-crossover recombination event at the *tcrB* locus was verified by colony PCR using a *tcrB*-specific primer pair. The resulting recombinant strains were designated p*_fim_*(239 b)-*cat*, p*_fim_*(151 b)-*cat*, p*_fim_*(109 b)-*cat*, p*_fim_*(59 b)-*cat* and p*_fim_*(33 b)-*cat*, respectively.

### Construction of the *fimR*-deficient Strain, the *fimR*-*fimA* Double Deficient Mutant and Complementation of *fimR*-deficiency

A 2.9-kbp amplicon containing *fimR* and its flanking region was generated by PCR using the primer pair fimR/S8023SpeI and fimR/AS10915SacI. The PCR product was subsequently cloned into the *Xba*I and *Sac*I sites of pSU21 [51]. The 19^th^ to 203^rd^ nt 3′ to the ATG start codon of *fimR* was deleted by inverse PCR with primers fimR/AS9576SmaI and fimR/S9761SmaI and replaced by an Em resistance gene (*erm*). The resulting plasmid was introduced into VT930, the wild-type p*_fim_*(445 b)*-cat* strain and its derivatives to inactivate *fimR* by allelic exchange. The correct inactivation was confirmed by colony PCR using a *fimR*-specific primer pair, and the resulting recombinant strains were designated VT930_Δ*fimR*, ΔR_p*_fim_*(445 b)-*cat*, ΔR_p*_fim_*(239 b)-*cat*, ΔR_p*_fim_*(151 b)-*cat*, ΔR_p*_fim_*(109 b)-*cat*, ΔR_p*_fim_*(59 b)-*cat*, and ΔR_p*_fim_*(33 b)-*cat*, respectively. To generate a *fimR* complementation strain, a DNA fragment containing the intact *fimR*, its 5′ flanking region of 340 bp, and its 3′ flanking region of 120 bp was generated by PCR using primers fimR/S9221BamHI and fimR/AS10331PstI. The product was subsequently cloned into the *E. coli*-streptococcal shuttle vector, pDL276 [52]. The identity of the PCR fragment was confirmed by sequencing analysis, and the correct chimeric plasmid (pHR6) was introduced into ΔR_p*_fim_*(445 b)-*cat* to generate strain Δ*fimR*/pHR6. The presence of pHR6 in the complementation strain was confirmed by plasmid isolation and restriction endonuclease analysis.

### CAT Assay

Mid-log phase cultures (optical density at 600 nm [OD_600_] = 0.6) grown in TH or FMC containing various amounts of metal ions were harvested, washed once with 10 mM Tris, pH 7.8, and resuspended in 2.5% of the original culture volume in the same buffer. Total protein lysates from concentrated cell suspensions were obtained as described previously [53]. The protein concentration was measured by Bio-Rad protein assay (United States) and bovine serum albumin (BSA) was served as the standard. CAT activities were determined by the method of Shaw [54], and the specific activities were calculated as nmole Cm acetylated min^−1^ (mg total protein) ^−1^.

### Purification of His-FimR and Preparation of Polyclonal Antiserum

The coding region of *fimR* was amplified from wild-type FW213 by PCR using primers fimR/MSSacI and fimR/stopASPstI. The amplicon was digested with *Sac*I and *Pst*I, and cloned into pQE30 (Qiagen, United States) at the compatible sites to generate pQE30/fimR. The identity of pQE30/fimR was confirmed by sequencing analysis. The induction and purification of His-FimR under native conditions was carried out according to the manufacturer’s instruction. Briefly, the *E. coli* strain harboring pQE30/fimR was grown to an OD_600_ of 0.2 initially, to which IPTG was added to a final concentration of 1 mM and the culture was incubated at 37°C for an additional hour to induce the expression of His-FimR. At the end of the induction, cells were collected, washed and lysed by French Press. His-FimR was purified from the total cell lysate by using the nickel-affinity chromatography. The bound protein was eluted with 250 mM imidazole. The identity of the purified His-FimR was further confirmed by Matrix-Assisted Laser Desorption/Ionization Time of Flight Mass Spectrometry (MALDI-TOF). For EMSA, the purified protein was dialyzed against 5 liter of buffer containing 10 mM Tris, pH 7.5, 1 mM DTT, 1 mM EDTA and 1% (v/v) glycerol, at 4°C for 16 h prior to use. To generate polyclonal anti-FimR antiserum in rabbits (Yao-Hong Biotechnology Inc., Taiwan), the isolated protein was first separated on 12% PAGE. The region containing His-FimR was excised and then used as an antigen. The specificity and titer of the antiserum were examined by Western blot analysis (Figure S3).

### EMSA

Two 53-mer oligos (pfimbox/S and pfimbox/AS) containing the complementary sequences of the 13^th^ to 65^th^ nt 5′ to the ATG of *fimC* were annealed and end-labeled with biotin using Biotin 3′ end DNA labeling kit (Pierce, United States). The binding reaction between the His-FimR and p*_fim_* probe was carried out in the presence of 0.1 mM MnCl_2_, 5 mM MgCl_2_, 50 mM KCl, 1 mM DTT, 5% (v/v) glycerol, 10 mM Tris (pH 7.5), 250 µg ml^−1^ BSA and 50 µg ml^−1^ poly(dI-dC). To each 20 µl binding reaction, 40 fmol labeled probe was used. Non-specific competition was carried out by including an internal fragment of *tcrB* (300 bp) without labeling in 10-fold excess in the reaction mixture. All reactions were incubated at room temperature for 20 min and then resolved on 6% native polyacrylamide gels. The DNA and protein complex was electro-transferred on to Nylon membranes, and detected by using Chemiluminescent nucleic acid detection module kit (Pierce).

### ChIP-qPCR

ChIP assay was performed by the method of Grainger et al. [55] with minor modifications. Briefly, the mid-exponential phase culture of *S. parasanguinis* FW213 in FMC supplemented with 0.01 or 50 µM MnCl_2_, 0.1 or 50 µM FeSO_4_, 1 mM MgSO_4_, and 1 mM CaCl_2_ was cross-linked with formaldehyde, washed, and then resuspended in 1/50 of the original culture volume in the lysis buffer [55]. The cell suspensions were subjected to mechanical disruption as described above, and the cellular DNA in the clear lysate was shared by sonication to generate DNA fragments with an average size of 0.5 to 1 kbp. Prior to precipitation with the antiserum, the DNA suspension was incubated first with A/G agarose (Merck Millipore, United States), salmon sperm DNA and BSA at 4°C for 1 h. The insoluble complexes were removed by centrifugation and an aliquot of the supernatant was used in immuoprecipitation reactions with the polyclonal anti-FimR antiserum. The negative control was carried out by using the pre-immunized rabbit serum, and the supernatant of this reaction was used as an input control. Immunoprecipitated samples were uncross-linked at 65°C for 12 h. DNA was then purified from the samples by phenol chloroform extraction and precipitation. 1/15 of the final product was then used in the qPCR analysis. The qPCR was carried out using the Power SYBR® Green PCR Master Mix and 7500 Fast real-time PCR system (Applied Biosystem, United States). The data were analyzed by using 7500 software v2.0.5. Each PCR reaction contains 250 nM of primers fimC/AS5025 and fimC_5′/S4731. Thermal cycler conditions were as follows: 95°C for 10 min followed by 40 cycles of 95°C for 15 sec and 60°C for 1 min. Each reaction was run in triplicates, and at least three samples were analyzed. Of note, a melting curve analysis was performed at the end of the amplification to ensure the amplification efficiency. The ΔCq of each sample was normalized with pre-immunized serum control and input control. As Mn^2+^ is a known cofactor for FimR, the ΔCq derived from the sample grown in 50 µM MnCl_2_ and 0.1 µM FeSO_4_ was used as the reference. The relative quantity of each sample was calculated as the ΔCq of the sample compared to the reference using the formula 2^ΔΔCq^.

### The Effect of Paraquat on Growth

To examine the sensitivity of *S. parasanguinis* to oxidative stressors, cultures at OD_600_ = 0.4 were diluted at 1∶50 in TH medium containing various amounts of paraquat. The growth was monitored at OD_600_ using a Bioscreen C growth monitor (Oy Growth Curves AB Ltd., Finland). Sterile mineral oil was added over the cell suspension to create a reduced oxygen environment, and the plate was shaken for 15 s prior to each reading. For each strain and condition, at least four samples were examined.

### Acid Killing

Cultures at OD_600_ = 0.4 were harvested, washed once with 0.1 M glycine buffer, pH 7, and then resuspended in 1/10 of the original culture volume in 0.1 M glycine buffer at pH 3. The viable counts of the bacterial suspension in pH 3 at 15, 30, and 45 min were determined by serial dilution and plating. The survival rate was expressed as a percentage of the viable count at each time point compared to the count prior to acid treatment. For each strain, at least three independent experiments were performed and all plating was done in triplicates.

### Macrophage Survival Assays

Human monocytic cell line, THP-1, and mouse RAW264.7 macrophages (Bioresource Collection and Research Center, Taiwan) were maintained in RPMI 1640 supplemented with 10% (v/v) heat-inactivated fetal calf serum and 2 mM L-glutamine. THP-1 cells (2×10^5^ ml^−1^) were activated by phorbol 12-myristate 13-acetate (PMA) at a final concentration of 1 µg ml^−1^ for two days before use. Mouse RAW264.7 macrophages (3×10^5^ ml^−1^) were allowed to adhere to plastic plates for 12 h prior to infection with bacteria. *S. parasanguinis* FW213 and its derivatives were grown to OD_600_ = 0.4, washed once with PBS and resuspended in RPMI1640 or IMDM (without serum) at 2∼8×10^8^ cells ml^−1^. All infections were done at a MOI of 100 for 1 h. At the end of infection, non-internalized bacteria were removed by washing twice with PBS and the remaining extracellular bacteria were killed by adding penicillin-gentamicin at a final concentration of 100 units ml^−1^ and 200 µg ml^−1^, respectively, followed by incubation at 37°C for 1 h. The culture medium was removed and washed twice with PBS to remove the residual antibiotic. The cells were lysed in PBS containing 0.1% Triton X-100 for 10 min. Bacterial counts in the cell lysates were then determined by serial dilutions and plating. The survival rate was calculated as a percentage of the recovered bacterial counts compared to the number of bacteria used in each infection.

## Supporting Information

Figure S1 **The CAT activities in wild-type** ***S. parasanguinis*** **FW213, Δ** ***fimR*** **, and the** ***fimR*** **complementation strain (Δ** ***fimR*** **/pHR6) harboring a single copy of p** ***_fim_*** **(445 b)** ***-cat*** **at the** ***tcrB*** **locus.** All strains were grown in TH to OD_600_ = 0.6. Values are means and standard deviations of three independent experiments.
(TIF)

Figure S2 **The activity of p** ***_fap1_*** **under various metal growth conditions.** *S. parasanguinis* FW213 harboring a single copy of p*_fap1_-cat* at the *tcrB* locus was cultivated in FMC containing 0.01 µM MnCl_2_ and 0.1 µM FeSO_4_ (I), 0.01 µM MnCl_2_ and 50 µM FeSO_4_ (II), 50 µM MnCl_2_ and 0.1 µM FeSO_4_ (III), 50 µM MnCl_2_ and 50 µM FeSO_4_ (IV). All cultures were supplemented with 1 mM MgSO_4_ and 1 mM CaCl_2_. Values are means and standard deviations of three independent experiments.
(TIF)

Figure S3 **Western analysis with the anti-FimR antiserum.** 25 µg of total cellular proteins prepared from wild-type *S. parasanguinis* FW213 (I) and the *fimR*-deficient strain (II) were separated on 12% SDS-PAGE, transferred to a piece of membrane and probed with the polyclonal antibody against FimR. The primary antibody was used at a dilution of 1∶200000 (A) and 1∶10000 (B), respectively, and the secondary antibody, goat anti-rabbit IgG, was used at 1∶10000. The molecular weight of FimR in kDa is indicated.
(TIF)
