# Supplementary figures and images for: The Expression of the fim Operon Is Crucial for the Survival of Streptococcus parasanguinis FW213 within Macrophages but Not Acid Tolerance
Source: PLoS One. 2013 Jun 18;8(6):e66163. doi: 10.1371/journal.pone.0066163 (PMC3688865; doi:10.1371/journal.pone.0066163)

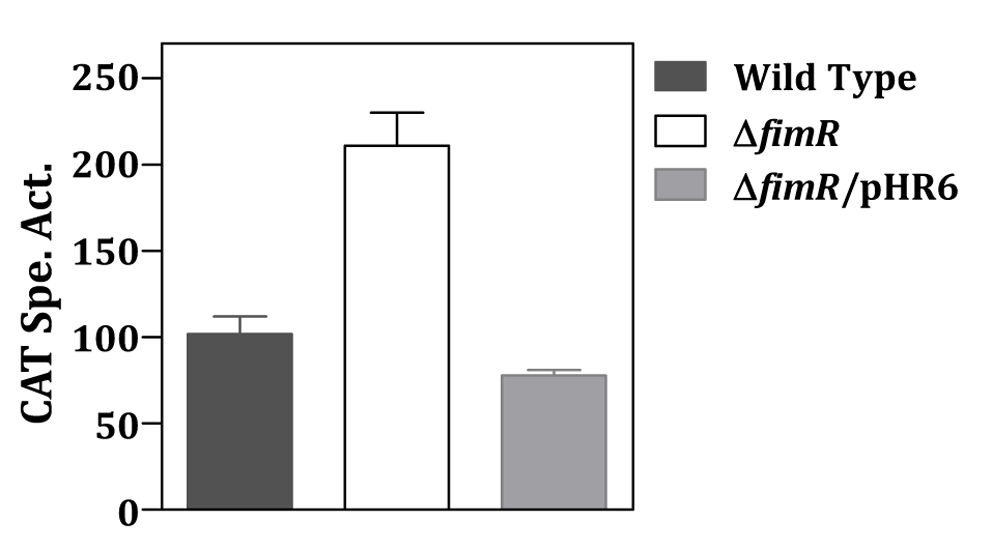

Supplement: Figure S1 — The CAT activities in wild-type S. parasanguinis FW213, Δ fimR , and the fimR complementation strain (Δ fimR /pHR6) harboring a single copy of p fim (445 b) -cat at the tcrB locus. All strains were grown in TH to OD600 = 0.6. Values are means and standard deviations of three independent experiments. (TIF) [file pone.0066163.s001.tif]

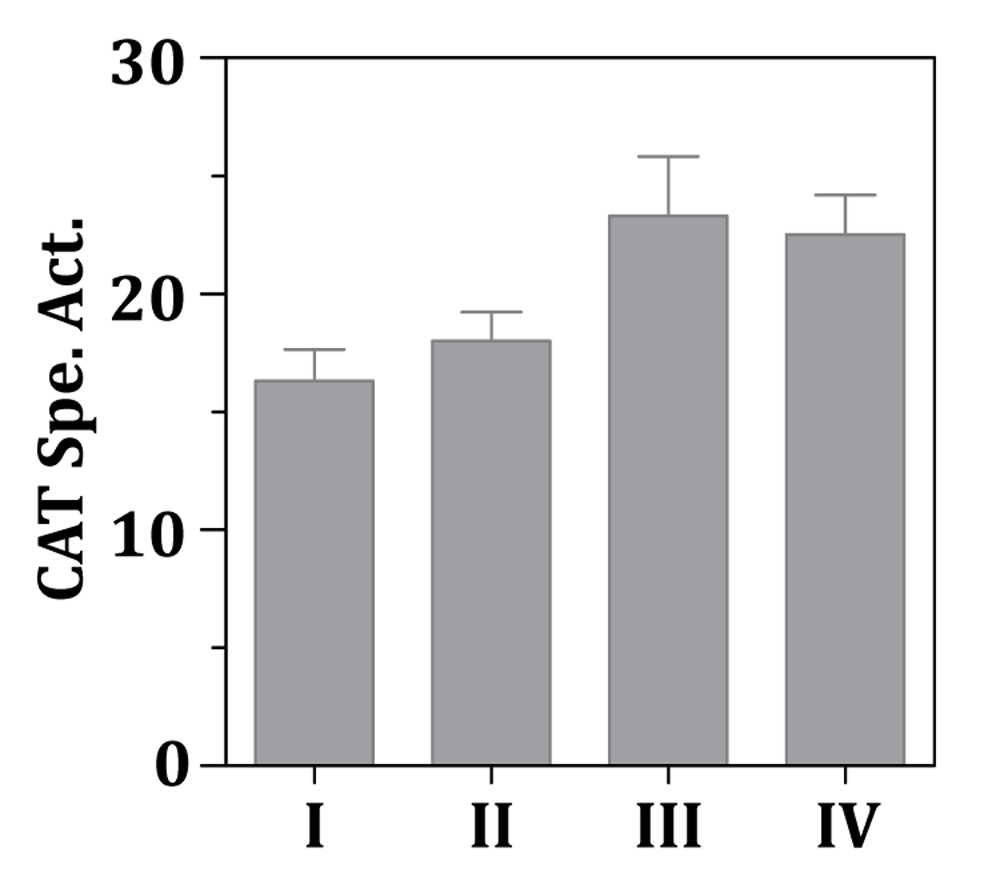

Supplement: Figure S2 — The activity of p fap1 under various metal growth conditions. S. parasanguinis FW213 harboring a single copy of pfap1-cat at the tcrB locus was cultivated in FMC containing 0.01 µM MnCl2 and 0.1 µM FeSO4 (I), 0.01 µM MnCl2 and 50 µM FeSO4 (II), 50 µM MnCl2 and 0.1 µM FeSO4 (III), 50 µM MnCl2 and 50 µM FeSO4 (IV). All cultures were supplemented with 1 mM MgSO4 and 1 mM CaCl2. Values are means and standard deviations of three independent experiments. (TIF) [file pone.0066163.s002.tif]

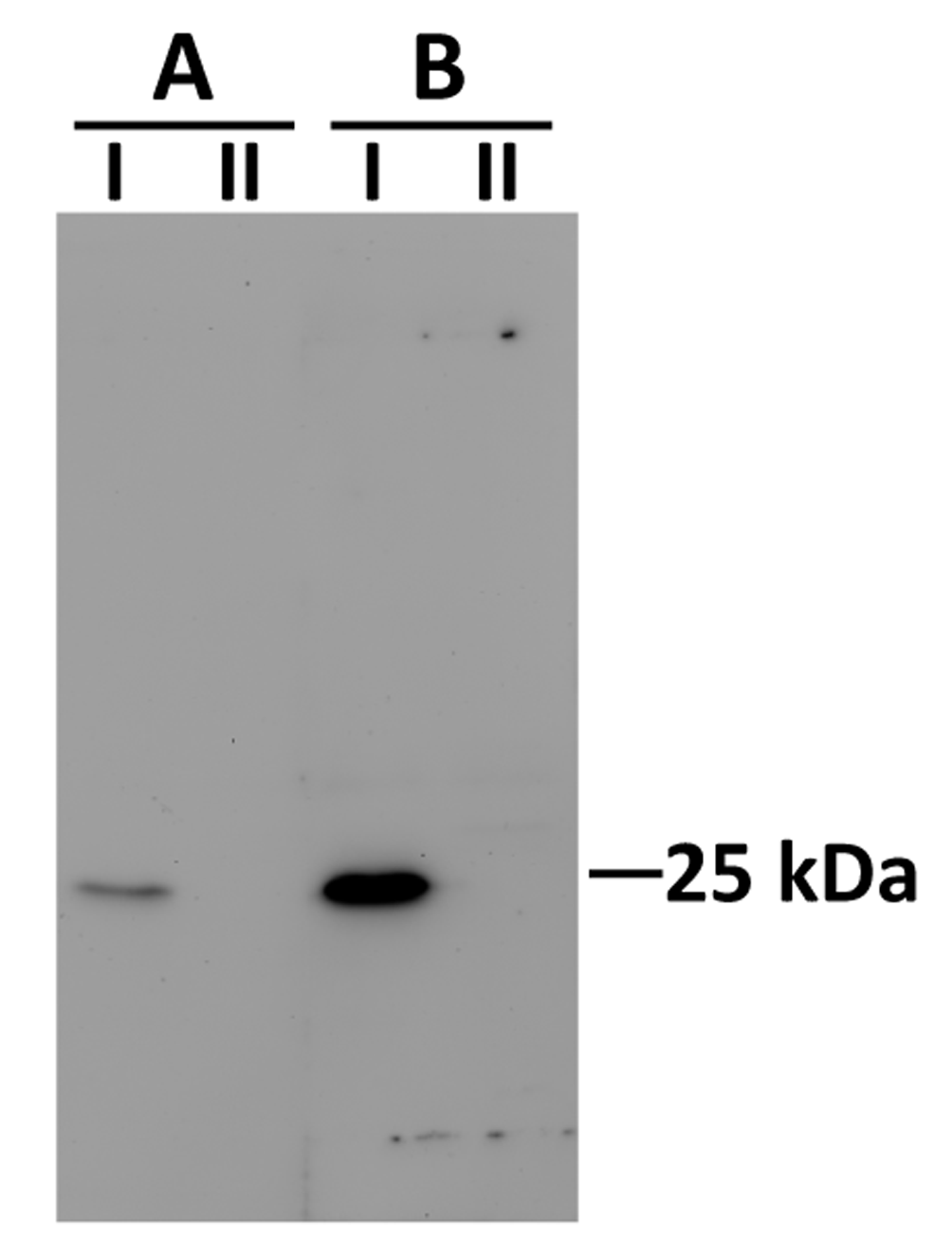

Supplement: Figure S3 — Western analysis with the anti-FimR antiserum. 25 µg of total cellular proteins prepared from wild-type S. parasanguinis FW213 (I) and the fimR-deficient strain (II) were separated on 12% SDS-PAGE, transferred to a piece of membrane and probed with the polyclonal antibody against FimR. The primary antibody was used at a dilution of 1∶200000 (A) and 1∶10000 (B), respectively, and the secondary antibody, goat anti-rabbit IgG, was used at 1∶10000. The molecular weight of FimR in kDa is indicated. (TIF) [file pone.0066163.s003.tif]
